# Pharmacological inhibition of CDK4/6 impairs diffuse pleural mesothelioma 3D spheroid growth and reduces viability of cisplatin-resistant cells

**DOI:** 10.3389/fonc.2024.1418951

**Published:** 2024-07-01

**Authors:** Aurora Costa, Iris Maria Forte, Francesca Pentimalli, Carmelina Antonella Iannuzzi, Luigi Alfano, Francesca Capone, Rosa Camerlingo, Alessandra Calabrese, Claudia von Arx, Reyes Benot Dominguez, Massimiliano Quintiliani, Michelino De Laurentiis, Andrea Morrione, Antonio Giordano

**Affiliations:** ^1^ Department of Medical Biotechnologies, University of Siena, Siena, Italy; ^2^ Experimental ClinicalOncology of Breast Unit, Department of Breast and Thoracic Oncology, Istituto Nazionale Tumori IRCCS “Fondazione G. Pascale”, Naples, Italy; ^3^ Department of Medicine and Surgery, LUM University “Giuseppe De Gennaro”, Bari, Italy; ^4^ Experimental Pharmacology Unit-Laboratories of Naples andMercogliano (AV), Istituto Nazionale Tumori IRCCS “Fondazione G. Pascale”, Naples, Italy; ^5^ Cell Biology and Biotherapy Unit, Istituto Nazionale Tumori, IRCCS, Fondazione G. Pascale, Naples, Italy; ^6^ Sbarro Institute for Cancer Research and Molecular Medicine, Center for Biotechnology, Department of Biology, College of Science and Technology, Temple University, Philadelphia, PA, United States; ^7^ SHRO Italia ETS, Candiolo, Italy

**Keywords:** diffuse pleural mesothelioma (DPM), cyclin-dependent kinases inhibitors (CDKi), cell cycle, precision medicine, cisplatin, synergism, apoptosis

## Abstract

**Introduction:**

Diffuse pleural mesothelioma (DPM) of the pleura is a highly aggressive and treatment-resistant cancer linked to asbestos exposure. Despite multimodal treatment, the prognosis for DPM patients remains very poor, with an average survival of 2 years from diagnosis. Cisplatin, a platinum-based chemotherapy drug, is commonly used in the treatment of DPM. However, the development of resistance to cisplatin significantly limits its effectiveness, highlighting the urgent need for alternative therapeutic strategies. New selective inhibitors of cyclin-dependent kinases 4 and 6 (CDK4/6) have shown promise in various malignancies by inhibiting cell cycle progression and suppressing tumor growth. Recent studies have indicated the potential of abemaciclib for DPM therapy, and a phase II clinical trial has shown preliminary encouraging results.

**Methods:**

Here, we tested abemaciclib, palbociclib, and ribociclib on a panel of DPM cell lines and non-tumor mesothelial(MET-5A) cells.

**Results:**

Specifically, we focused on abemaciclib, which was the mosteffective cytotoxic agent on all the DPM cell lines tested. Abemaciclib reduced DPM cell viability, clonogenic potential, and ability to grow as three-dimensional (3D) spheroids. In addition, abemaciclib induced prolonged effects, thereby impairing second-generation sphere formation and inducing G0/G1 arrest and apoptosis/ necrosis. Interestingly, single silencing of RB family members did not impair cell response to abemaciclib, suggesting that they likely complement each other in triggering abemaciclib’s cytostatic effect. Interestingly, abemaciclib reduced the phosphorylation of AKT, which is hyperactive in DPM and synergized with the pharmacological AKT inhibitor (AKTi VIII). Abemaciclib also synergized with cisplatin and reduced the viability of DPM cells with acquired resistance to cisplatin.

**Discussion:**

Overall, our results suggest that CDK4/6 inhibitors alone or in combination with standard of care should be further explored for DPM therapy.

## 1 Introduction

Diffuse pleural mesothelioma (DPM) of the pleura is an asbestos-related cancer with a poor prognosis for which limited treatment strategies exist to date (1). DPMs present as three main histological subtypes, epithelioid, sarcomatoid, and biphasic, characterized by epithelial cells, spindle-shaped cells, or both cell types, respectively (2, 3). Sarcomatoid tumors have the worst prognosis (3). Since 2003, the combination of pemetrexed and platinum compounds has been the standard first-line therapy (4), until the recent introduction of immune checkpoint inhibitors (ICIs) (5, 6), whose use is still under discussion for high costs, toxicity, and efficacy. Second-line treatment options, including vinorelbine or gemcitabine monotherapy, ICIs, or rechallenge with platinum–pemetrexed doublet, also show limited efficacy (7). Despite having a predominant etiology linked to asbestos exposure, inherited heterozygous germline mutations of the deubiquitylase BRCA-associated protein 1 (BAP1) are associated with a higher incidence of DPM in some families (8). DPM is highly heterogeneous at the molecular level, which is a key hurdle in developing effective therapies (9). The identification of common chromosomal losses has, in recent years, significantly transformed DPM diagnosis (1, 9), which now involves testing for the inactivation of tumor suppressor genes, in particular the loss of p16INK4A-p14ARF encoded by the *CDKN2A* locus, which is assessed by fluorescence *in situ* hybridization (FISH) (or methylthioadenosine phosphorylase (MTAP) immunohistochemistry), neurofibromatosis type 2 (*NF2*), and *BAP-1*, which is inactivated at somatic levels in more than 60% of sporadic DPM (10–13). However, DPM is still diagnosed at a late stage, and biomarkers of earlier diagnosis are still eagerly required.

Multiple targets and pathways of interest have been identified from genomic studies of DPM (14), but the clinical translation of targeted approaches has been hampered by various challenges, including a highly immunosuppressive environment (15) and the emergence of drug resistance (16).

*CDKN2A* deletion has been documented in approximately 40% of DPM cases and up to 90% of DPM cell lines (17). *CDKN2A* encodes for both p16INK4A and p14ARF and causes the functional inactivation of the key tumor suppressors controlling cell cycle progression, such as RB1 and p53 (18). The RB family of proteins, also known as pocket proteins, are key cell cycle regulators and are positioned at the crossroads of multiple pathways underlying cell fate decisions. RB inactivation, either direct or indirect, is a hallmark of most tumors, including DPM. The role of RB family proteins as gatekeepers of the G1/S cell cycle transition has been well characterized (19, 20). This RB function is mainly regulated through phosphorylation mediated by cyclin-dependent kinase (CDK) complexes, which interfere with the RB binding to the E2F family transcription factors, which regulate the expression of genes necessary for cell cycle progression. Many efforts have been made to translate RB-based strategies to the clinical setting (21, 22), and strategies aimed at restoring the RB canonical cell cycle restraining function through new-generation selective cyclin-dependent kinases 4 and 6 inhibitors (CDK4/6i) have been successfully translated the clinical practice (20).

Indeed, this novel class of CDK4/6i has been used in the treatment of patients with HR+, HER2− advanced breast cancer, either as first-line therapy in combination with aromatase inhibitors or as second-line therapy in combination with fulvestrant (23–25). Their use has been also approved for early breast cancer, and numerous clinical trials are ongoing to study their potential in other cancers. These new-generation CDK4/6i include three compounds commercially called ribociclib, palbociclib, and abemaciclib. Previous studies have investigated their efficacy in preclinical models of DPM (26–28), proving their ability to counteract malignant features by inhibiting CDK4/6 activity, which correlates with worse overall survival (27). These studies confirmed the expected mechanism of action whereby CDK4/6i reinstate RB1 cytostatic activity in DPM defective for *CDKN2A*, whereas intrinsically insensitive tumors show high p16/*CDKN2A* expression, which impairs the activating CDK4 T172 phosphorylation (25). Interestingly, CDK4/6i synergize also with *i*) cisplatin and pemetrexed (29, 30), which is DPM standard-of-care; *ii*) PI3K/mTOR inhibitors (23); *iii*) mir-206, which targets CDK6 (31); *iv*) immunotherapy with anti-PD1 agents (nivolumab) (32); and *v*) auranofin, which targets Trx2 and the antioxidant response (29). Encouraging results were obtained in the MiST2 single-arm, open-label, phase 2 clinical trial (NCT03654833), testing abemaciclib in chemotherapy-treated patients with p16-negative DPM, which met its primary endpoint of disease control rate (complete or partial response, or stable disease) at 12 weeks. Despite overall promising results, these studies also showed that intrinsic or acquired resistance can arise and that optimal combinatorial strategies and sequence of treatments need to be carefully evaluated to promote definitive cell cycle exit rather than malignant cell quiescence, which can potentially lead to escape from cytotoxic therapies.

Our previous research showed that RBL2, a member of the RB family, plays an important role in inducing DPM cell apoptosis following inhibition of the AKT oncogenic pathway, and roscovitine, a first-generation CDK inhibitor, synergized with AKT inhibition in DPM cells (33).

Therefore, in this study, we first wanted to determine the half-maximal inhibitory concentration (IC50) values of these new-generation CDK4/6i on our panel of DPM cell lines. Then, we tested these drugs for their ability to affect growth as three-dimensional (3D) spheroids, which better recapitulate cancer features and are useful preclinical models to study antitumoral drug response (34). Additionally, we evaluated whether depletion of RB family members counteracted abemaciclib’s effect on cell viability and determined whether abemaciclib (like roscovitine) synergizes with the selective AKT inhibitor (AKTi VIII). Lastly, we tested whether abemaciclib can overcome resistance to cisplatin in a model of resistant MSTO-211H cells.

## 2 Materials and methods

### 2.1 Cell lines

NCI-H2452, MMB, ISTMES-2 (representative of epithelioid histotypes), MSTO-211H (biphasic type), NCI-H28, and NCI-H2052 (derived from sarcomatoid type) mesothelioma cell lines and MET-5A immortalized normal mesothelial cells were purchased from American Type Culture Collection (ATCC). ISTMES-2 was purchased from the istituto nazionale per la ricerca sul cancro di genova (ISTGE) cell repository. NCI-H28, NCI-H2452, MSTO-211H, and NCI-H2052 cells were maintained in RPMI-1640 supplemented with 10% fetal bovine serum (FBS), 1% penicillin-streptomycin, and 1% glutamine. ISTMES-2 was grown in Dulbecco’s modified Eagle’s medium (DMEM) and MMB in F12 both supplemented with 10% FBS, 1% penicillin-streptomycin, and 1% glutamine. MET-5A cells were grown in Medium 199 with 10% FBS, 0.5% penicillin-streptomycin, 1% glutamine, 3.3 nM epidermal growth factor, 400 nM hydrocortisone, and 870 nM insulin. All cell culture reagents were purchased from Sigma-Aldrich Corp. (St. Louis, MO, USA). To generate RB1/p105-, RBL1/p107-, and RBL2/p130-depleted cells, HEK-293FT cells were transfected with PAX2 packaging plasmid, PMD2G envelope plasmid, and pLKO.1. The following pLKO.1 vectors were used: Broad Institute clone TRCN0000040163, TRCN0000040018, and TRCN0000039923 expressing shRNA targeting the human *RB1*, *RBL1*, and *RBL2* mRNA, respectively, or a scrambled shRNA (pLKO.1 shSCR, gift from S. Stewart, Addgene plasmid #17920). Following transfection, supernatants were collected, filtered, and used for transducing DPM cells. Three days post-infection, cells were selected with 2 μg/mL puromycin (Sigma-Aldrich).

To generate cisplatin-resistant cell lines, MSTO-211H and NCI-H2052 cells were treated for five cycles with increased concentrations of cisplatin (*Calbiochem*) from 5 μM, 10 μM, 15 μM, and 20 μM to 25 μM, every time for 72 h, and then grown in drug-free medium for 10 days (35). Each treatment was not administered until the cells were in exponential growth phase. The cells were maintained in RPMI-1640 supplemented with 20 μM cisplatin (approximately IC50) (36), 10% FBS, 1% penicillin-streptomycin, and 1% glutamine.

### 2.2 Cell viability assay

DPM cell lines, immortalized normal mesothelial MET-5A cells, and MSTO-211H depleted of retinoblastoma proteins (RB1/p105, RBL1/p107, and RBL2/p130) or expressing shRNA scramble as control were seeded in triplicates in 96-well plates at a density of approximately 1,000 cells/well and allowed to attach for 24 h. Cells were then treated with palbociclib, ribociclib, and abemaciclib (all purchased from Selleckchem, Houston, TX, USA) at doses ranging from 1.56 µM to 50 µM for 72 h. At the end of the treatment, cell viability was evaluated by MTS assay (CellTiter 96^®^ AQueous One Solution Cell Proliferation Assay, Promega, Milan, Italy), following the manufacturer’s instructions. The percentage of cell viability was calculated using 100% as cell viability of untreated cells. The CDKi IC50 was determined by a dose–response curve using GraphPad Prism 7 Software.

MSTO-211H and NCI-H2052 cisplatin-resistant cells were seeded in triplicate as indicated above and then treated with abemaciclib at doses ranging from 2 µM to 32 µM for 72 h. At the end of the treatment, cell viability was evaluated by MTS assay.

### 2.3 Clonogenic assay

For the clonogenic assay, 200 cells were seeded in 6-well plates and, after 24 h, treated with abemaciclib for 72 h at IC50 for each cell line. After 10 days, colonies were fixed with methanol and stained at room temperature for 30 min with crystal violet (Sigma-Aldrich).

### 2.4 Spheroid generation

Spheroid formation was performed in cells grown in RPMI-1640 supplemented with 10% FBS, 1% penicillin-streptomycin, and 1% glutamine (NCI-H28, NCI-H2452, MSTO-211H, and NCI-H2052). Spheroid formation was performed with two different strategies following two protocols: 1) The Single Spheroid Protocol and 2) the Multiple Spheroids Protocol [adapted from Campioni et al. (37)]. For both strategies, spheroids were freshly prepared from attached cell cultures 3 days (72 h) before each experiment. On the day of seeding (day 0), cells were detached with trypsin-EDTA and centrifuged at 290 ×*g* for 5 min. Next, the pellet was suspended in RPMI-1640 medium (with supplements), and cells were counted with a Bürker chamber. After that, the cells were seeded in U-bottom Ultra Low Attachment (ULA) 96-well microplates (Corning B.V. Life Sciences, Amsterdam, The Netherlands). To obtain single spheroids (protocol 1), cells were seeded at a density of 0.5 × 10^4^ cells/well in 100 µL/well. After seeding, the U-bottom ULA 96-well microplates were centrifuged at 340 ×*g* for 30 min to foster cell aggregation. Next, the microplates were incubated for up to 4 days at 37°C in a humidified atmosphere containing 5% CO_2_ during spheroid formation. To obtain multiple spheroids (protocol 2), cells were seeded at a density of 3 × 10^4^ cells/well in 2 mL/well into ULA 6-well plates (Corning B.V. Life Sciences, Amsterdam, The Netherlands). During spheroid development, the 6-well plates were incubated for up to 5 days at 37°C in a humidified environment containing 5% CO_2_.

#### 2.4.1 Second-generation spheroid protocol

After multiple spheroids were created (day 5), they were broken using trypsin-EDTA for 5/10 min, gently pipetted to separate single cells, and centrifuged for 5 min at 290 ×*g*. After suspending the pellet in RPMI-1640 medium (with supplements), the cells were counted and subsequently plated in ULA 6-well plates at a density of 3 × 10^4^ cells/well in 2 mL/well (Corning B.V. Life Sciences, Amsterdam, The Netherlands). During spheroid growth, the plates were incubated for up to 4 days at 37°C in a humidified atmosphere containing 5% CO_2_ (34).

### 2.5 Western blotting analysis

For total protein extraction, cells were lysed on ice for 30 min in 50 mM Tris-HCl pH 7.5, 1 mM EDTA pH 8.0, 150 mM NaCl, and 1% NP-40 buffer, supplemented with protease and phosphatase inhibitor cocktails (Roche, Basel, Switzerland). Protein samples were resolved in sodium dodecyl sulfate–polyacrylamide gel electrophoresis (SDS-PAGE) and blotted onto nitrocellulose membranes, which were then incubated with antibodies against RBL2/p130 (Abcam, Cambridge, UK; cat# ab183039), phosphoRBL2/p130 S941 (custom by Covalab, Villeurbanne, France), phosphoRBL2/p130 S639 (Santa Cruz, Dallas, TX, USA; cat# sc16301), RBL1/p107 (Cell Signaling Technology, Danvers, MA, USA), phosphoRBL1/p107 S975 (MyBioSource, San Diego, CA, USA; cat# MBS9214765), RB1/p105 (Cell Signaling, cat# 9309), phosphoRB1/p105 S780 (Cell Signaling, cat# 9308), AKT(pan)(C67E7) (Cell Signaling, cat# 4691), phosphoAKT S473 (Cell Signaling, cat# 9271), p27 (Abcam, cat# Ab32034), phospho-p70 S6 kinase (Th389) (Cell Signaling, #9205), S6K1 (Abcam, ab32529), and GAPDH (Santa Cruz, cat# sc-25778). After incubation with horseradish peroxidase-conjugated secondary antibodies, signals were detected through ECL (Amersham Biosciences, GE Healthcare, Little Chalfont, UK). Images were analyzed using ImageQuant LAS 500 (GE Healthcare, Little Chalfont, UK).

### 2.6 Cytofluorimetric analysis of cell cycle profile and cell death

For cell cycle analysis, all cell lines were treated with abemaciclib at IC50 value, collected after 72 h, washed with phosphate-buffered saline (PBS), and fixed in 70% ice-cold ethanol. Cells were then incubated at 37°C for 1 h with 50 μg/mL propidium iodide (PI; cat# P4170; Sigma-Aldrich) and 20 μg/mL RNase (cat# 9001-99-4; Sigma-Aldrich) and analyzed using BD FACSDiva Software 8.0 (BD Biosciences, San Jose, CA, USA). For apoptosis detection, cells were stained with Annexin V-FITC and PI (cat# 130-092-052; Annexin V-FITC kit; Miltenyi Biotec Inc., Bologna, Italy) according to the manufacturer’s instructions and analyzed using FACS (BD FACSCalibur, BD Biosciences).

### 2.7 Drug combination studies

DPM cells were treated for 72 h with abemaciclib and cisplatin and with abemaciclib and AKTi VIII both alone and in combination at various concentrations in a constant ratio, and cell viability was assessed using MTS assay. Synergism, additivity, or antagonism was determined through isobologram analysis using the ComboSyn software 1.0 (ComboSyn, Inc. Paramus, NJ, USA). Combination index (CI) values were also calculated according to the Chou–Talalay equation, using the CompuSyn software. CI < 1 indicates synergism, CI = 1 additivity, and CI > 1 antagonism. The r value represents the linear correlation coefficient of the median effect plot, which indicates the conformity of the data to the mass action law.

### 2.8 Statistical analysis

Statistical analyses were performed using Prism 7 (GraphPad software). Results were expressed as means ± standard deviation and derived from at least two independent experiments.

## 3 Results

### 3.1 New-generation cyclin-dependent kinase 4/6 inhibitors affect cell viability and clonogenic potential of DPM cell lines

**Figure 1:**
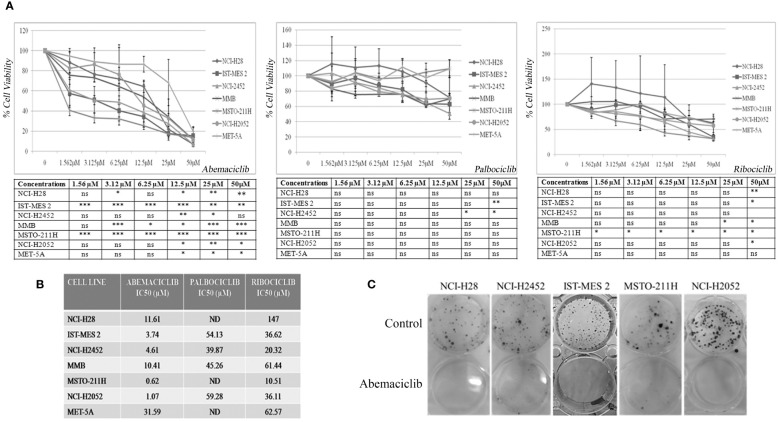
CDK4/6 inhibitors affect cell viability and colony formation of mesothelioma cell lines. **(A)** Data represent dose–response curves reporting the mean ± standard deviation of the effects of different doses of abemaciclib, palbociclib, and ribociclib on cell viability evaluated by MTS assay after 72 h of treatment in at least two experiments on DPM cell lines (NCI-H28, ISTMES-2, NCI-H2452, DPMB, MSTO-211H, and NCI-H2052) and immortalized normal mesothelial cells MET-5A. Results are expressed as percentage of cell viability (calculated with respect to control cells treated with DMSO alone). Statistically significant differences between each dose versus control, reported in tables, were evaluated by one-way repeated-measures ANOVA with Tukey’s post-test and indicated as follows: * p < 0.05; ** p < 0.01; *** p < 0.001. **(B)** IC50 values for each cell line. IC50 values for some cell lines were not determined (ND) or higher than the maximum dose used. The IC50 values were calculated using GraphPad Prism 5.01. **(C)** Long-term abemaciclib effects were assessed by clonogenic assay. Colonies were stained with crystal violet 10 days after a 72-h treatment with abemaciclib (IC50). Representative plates of three independent experiments are shown. DPM, diffuse pleural mesothelioma; DMSO, dimethyl sulfoxide. ns, not statistically significant.

We initially tested the activity of three new-generation CDKi, palbociclib, ribociclib, and abemaciclib, on a panel of DPM cell lines to determine their efficacy and IC50 values. Specifically, we treated NCI-H28, ISTMES-2, NCI-H2452, MMB, MSTO-211H, and NCI-H2052, which are representative of the three main DPM histotypes, with increasing concentrations of CDKi ranging from 1.56 µM to 50 µM, and we evaluated cell viability by MTS assay after 24 h, 48 h (data not shown), and 72 h of treatment (**Figure 1A**). In addition, we tested CDKi cytotoxicity on MET-5A, a normal mesothelium cell line, immortalized with the early region of the SV40 virus and therefore bearing inactive RB (**Figure 1A**). We determined for most of the cell lines the IC50 values, which are reported in **Figure 1B**. Compared with ribociclib and palbociclib, abemaciclib showed the highest cytotoxic effect on all the DPM cell lines tested, including NCI-H2052 and NCI-H28, which are derived from the most aggressive sarcomatoid histotype. Significantly, abemaciclib did not show comparable toxic effects on the control mesothelial cell line, MET-5A. With abemaciclib the most effective CDKi on our panel of DPM cells, we focused on its use and action in all subsequent experiments.

To verify whether abemaciclib exerted long-term cell growth inhibition, we performed clonogenic assays upon treatment at IC50 values of NCI-H28, NCI-H2452, ISTMES-2, MSTO-211H, and NCI-H2052, and we demonstrated that the CDKi dramatically reduced colony formation in all the DPM cell lines tested (**Figure 1C**). MET-5A did not form clones at the low cell density required for this assay, and the effect of abemaciclib was not therefore assessed in this cell line.

### 3.2 Abemaciclib inhibits malignant pleural mesothelioma spheroid formation

**Figure 2:**
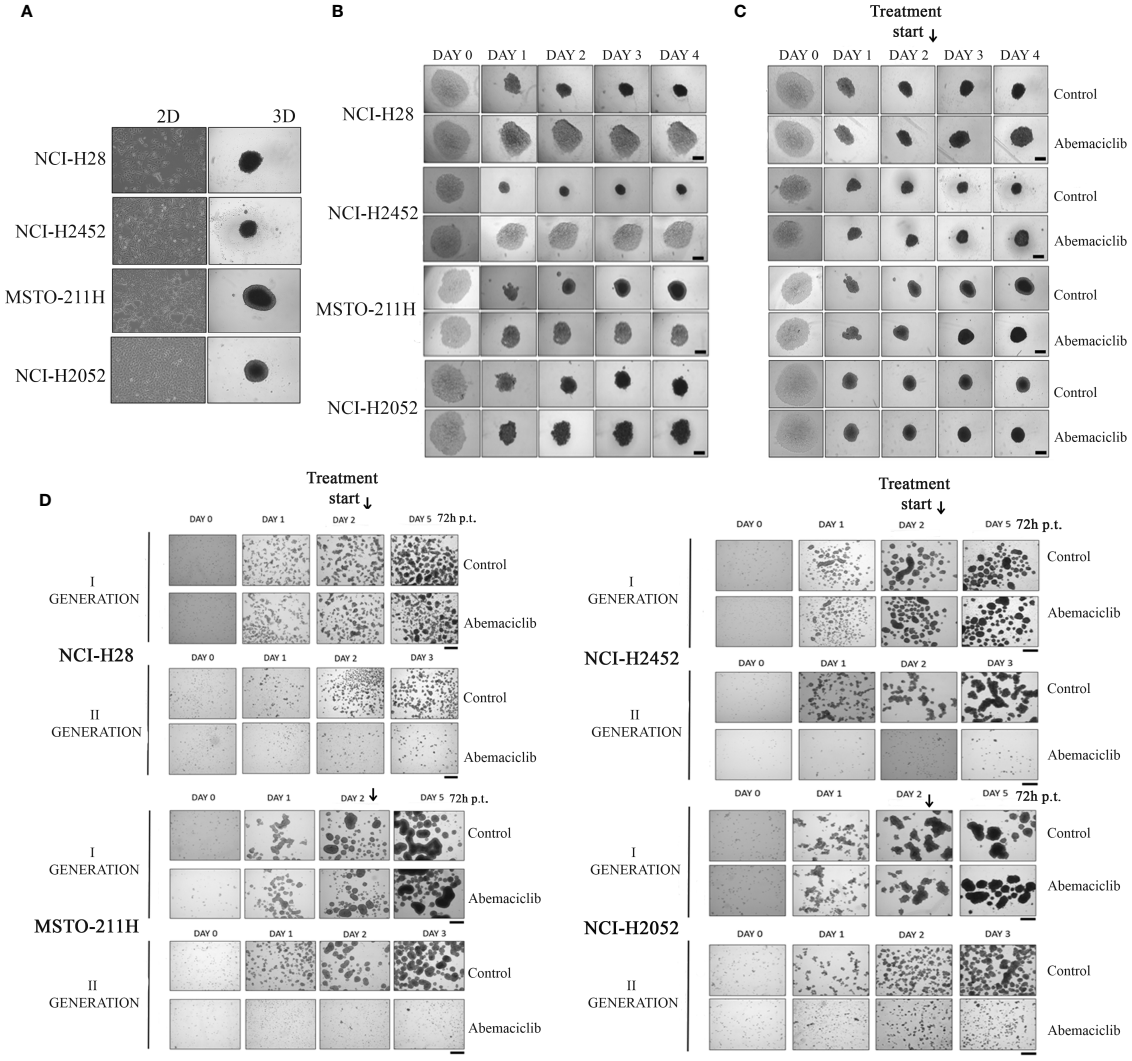
Effect of abemaciclib on mesothelioma 3D cultures. **(A)** DPM cell lines (NCI-H28, NCI-H2452, MSTO-211H, and NCI-H2052) are represented as 2D monolayer (left) and 3D single spheroid. Images were acquired 4 days after seeding. **(B)** DPM single spheroids were treated with abemaciclib on day 0. Cells were treated with abemaciclib (5 µM) or DMSO, as a control, starting on day 0. Images were acquired on days 0, 1, 2, 3, and 4. Abemaciclib at low concentration (5 µM) significantly inhibited the ability of DPM cells to generate spheroids. All experiments were performed at least three times. **(C)** DPM 2-day-old spheroids were treated with abemaciclib. Cells were seeded at a density of 0.5 × 10^4^ cells/well in U-bottom ULA 96-well plates and after 2 days were treated with abemaciclib (10 µM) or DMSO as control. Images were acquired on days 0, 1, 2 (day of treatment), 3, and 4. Experiments were performed at least three times. **(D)** Cells were seeded at a density of 30 × 10^4^ cells/well in ULA 6-well plates and after 2 days treated for 72 h with abemaciclib or DMSO as control. Spheroids were disaggregated in single cells and seeded again. Images were acquired on days 0, 1, and 2 (day of treatment); day 5 for first-generation spheroids; and days 1, 2, and 3 for second-generation spheroids. All experiments were performed at least three times. DPM, diffuse pleural mesothelioma; DMSO, dimethyl sulfoxide.

Three-dimensional cell culture systems represent preclinical models that better recapitulate patient tumor features and could reduce or, ideally, replace the use of animal models, thereby resolving the associated ethical and cost issues (34). We then tested the ability of abemaciclib to affect DPM cell lines grown as 3D spheroids. To this aim, we used ultra-low attachment (ULA) 96-well round-bottom plates, which favor the formation of centrally located single spheroids and, unlike conventional approaches, do not require coating to inhibit cell adhesion. We evaluated the capacity of four DPM cell lines (NCI-H28, NCI-H2452, MSTO-211H, and NCI-H2052) to grow as spheroids after 4 days (**Figure 2A**) and demonstrated that all cell lines formed single spheroids without the addition of any supplements. Notably, abemaciclib at 5-µM concentration significantly inhibited the ability of DPM cells to generate spheroids (**Figure 2B**). Because abemaciclib successfully blocked the formation of spheroids, we studied its impact on 2-day-old spheroids in order to determine whether the drug reached sufficient concentrations within the target cancer cell population to induce antitumoral effects. Two days after cell seeding, we treated fully developed single DPM spheroids with 5 µM abemaciclib for an additional 48 h. In the presence of abemaciclib, spheroids generated from NCI-H2452 of epithelioid histotype and NCI-H28 (reported as sarcomatoid on Cellosaurus but often as epithelioid in the literature) lost their integrity and were not as compact as compared to spheroids growing in media without the inhibitor (**Figure 2C**). In contrast, spheroids formed by biphasic (MSTO-211H) and sarcomatoid (NCI-H2052) histotypes showed a drastic increase in the necrotic core and an overall volume decrease, and the proliferative area completely disappeared (**Figure 2C**). Finally, to confirm the ability of abemaciclib to penetrate deeper into 3D cell structures and reach cells within the multicellular spheroid, we treated formed spheroids for 72 h (day 2) with 5 µM abemaciclib at the same concentration able to block the formation of first-generation spheroids. We then trypsinized the spheroids and seeded cells again to obtain second-generation spheroids. Our data demonstrated that abemaciclib affected all first-generation DPM spheroid cells (**Figure 2D**), significantly inhibiting their ability to form second-generation spheroids.

### 3.3 Abemaciclib treatment induces modulation of cell cycle-related protein levels in malignant pleural mesothelioma cells

To elucidate the molecular mechanisms underlying abemaciclib-dependent effects, we tested the phosphorylation and expression level of several cell cycle-related proteins, which are targets of CDK 4/6 complexes inhibited by the drug, in NCI-H28, NCI-H2452, ISTMES-2, MSTO-211H, NCI-H2052, and immortalized mesothelium cell line, MET-5A.

**Figure 3:**
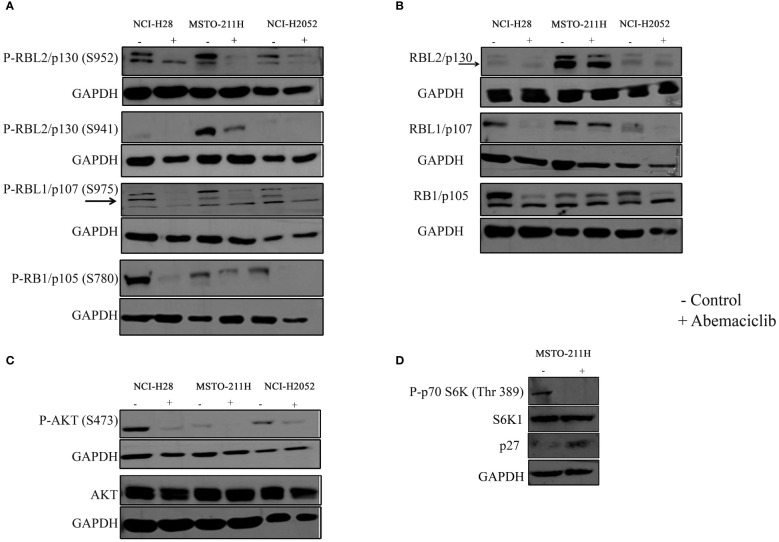
Effects of abemaciclib on Rb protein family. NCI-H28, MSTO-211H, and NCI-H2052 cells were treated according to their respective IC50 value for 48 h with abemaciclib or DMSO as a control. **(A)** Analysis of phosphorylated levels of RBL2/p130, RBL1/p107, and RB1/p105 proteins were determined by immunoblot. Arrows indicate bands corresponding to phosphorylated RBL1/p107 (S975). GAPDH was used as loading control. **(B)** Analysis of total levels of RBL2/p130, RBL1/p107, and RB1/p105 proteins determined by immunoblot. Arrows indicate bands corresponding to total RBL2/p130. GAPDH was used as loading control. **(C)** Levels of total and phosphorylated AKT were determined by immunoblot. GAPDH was used as normalization control. **(D)** Levels of phpspho-p70S6K (Thr 389), total S6K1 and p27 were determined by immunoblot in MSTO-211H cells upon abemaciclib treatment at its IC50 value. GAPDH was used as normalization control.

Thus, we tested by immunoblot basal and phosphorylation status of RB1/p105, RBL1/p107, and RBL2/p130 (**Figures 3A, B**). The expression of RB1/p105 and RBL1/p107 was dramatically decreased in NCI-H28 and NCI-H2052 cell lines, but not significantly affected in MSTO-211H cells (**Figure 3B**). The levels of phosphorylated pRB1/pp105 (S780) and pRBL1/pp107 (S975) proteins were sharply reduced in all DPM cell lines (**Figure 3A**; **Supplementary Figure 1A**). The total levels of RBL2/p130 were not altered by abemaciclib treatment, while the level of RBL2/p130 phosphorylation at S952 was significantly decreased (**Figures 3A, B**). These results suggest that abemaciclib differentially affects the expression and activation of RB protein family members. Accordingly, abemaciclib treatment reduced RB protein phosphorylation, thereby restoring their tumor-suppressive function.

It was previously demonstrated that abemaciclib inactivates AKT kinase in lung cancer (38). We recently demonstrated that in DPM cells, RBL2/p130 is a direct target of AKT, which mediated RBL2 phosphorylation at S941 (33). Notably, RBL2 was essential for apoptosis associated with AKT inhibition. To further define the mechanism of abemaciclib action, we then examined whether the AKT pathway was affected by CDK4/6 inhibitors treatment and demonstrated that abemaciclib effectively inhibited the AKT pathway after 48 h of treatment, as shown by the significant reduction of phosphorylated AKT (S473) levels in all DPM cells (**Figure 3C**). Accordingly, we observed a reduction of AKT-mediated RBL2/p130 S941 phosphorylation and an associated increase in the protein levels of the cell cycle inhibitor p27, another well-established AKT target (**Figure 3D**). We also observed that abemaciclib, by reducing AKT phosphorylation, also affected the phosphorylation levels of the p70S6K (Thr389), a kinase that is downstream mTOR signaling and affects tumorigenesis by acting on a variety of targets (**Figure 3D**).

### 3.4 Role of pocket proteins in MSTO-211H cell line response to abemaciclib

**Figure 4:**
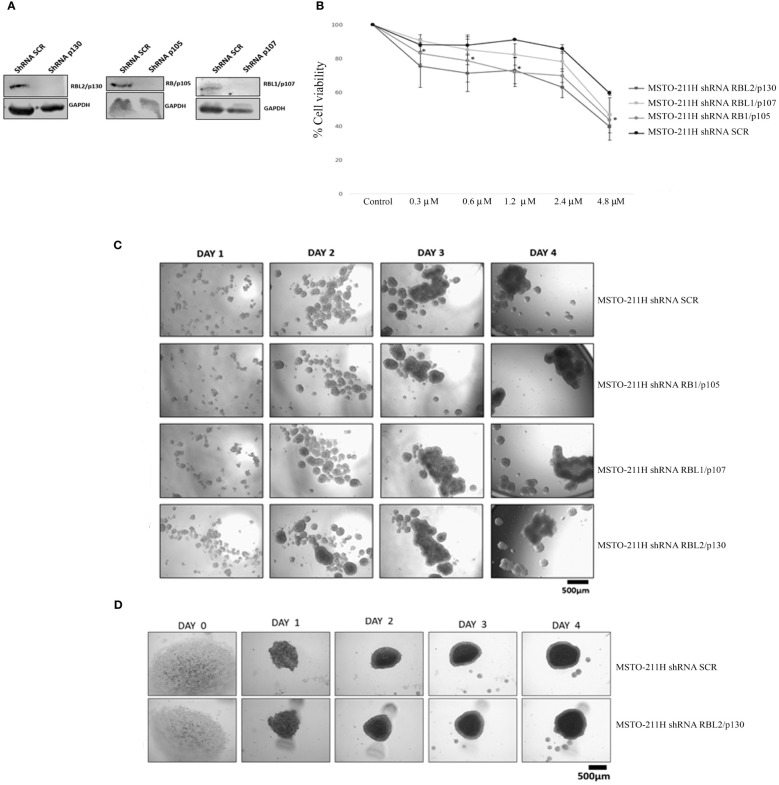
RB protein family depletion in MSTO-211H cells and abemaciclib response. **(A)** Western blotting of MSTO-211H cells depleted in each pocket protein generated by shRNA approaches. Expression was assessed by immunoblot with specific antibodies. ShRNASCR are sh-control-transfected cells. **(B)** The graph represents dose–response growth curves with different doses of abemaciclib (0.3 µM, 0.6 µM, 1.2 µM, 2.4 µM, and 4.8 µM) evaluated by MTS assay after 72 h of treatment in pocket protein-depleted MSTO-211H cells. Statistical analysis was carried out by unpaired Student’s t-test. Statistical significance was settled at *p < 0.05, **p < 0.005 vs. control. Data are presented as mean ± standard deviation expressed as percentages of cell viability (calculated with respect to control cells treated with DMSO alone) of three independent experiments (n = 3). **(C)** The image represents 4 days’ time-lapse of spheroids with pocket protein depletion in MSTO-211H cells. Cells were seeded at a density of 30 × 10^4^ cells/well in 3 mL of RPMI medium (+10% FBS and +1% l-Glu) in ULA 6-well plates. Images were acquired on days 0, 1, 2, 3, and 4. **(D)** Cells were seeded at a density of 0.5 × 10^4^ cells/well in U-bottom ULA 96-well plates in 100 μL of RPMI medium (+10% FBS and +1% l-Glu). Images were acquired on days 0, 1, 2, 3, and 4. Scale bar: 500 μm. All experiments were performed at least three times. DMSO, dimethyl sulfoxide; FBS, fetal bovine serum.

In order to better characterize the role of the RB protein family upon abemaciclib treatment, we generated MSTO-211H cells stably depleted of *RB1*, *RBL1*, and *RBL2* by lentiviral-based shRNA approaches (**Figure 4A**) and analyzed cell viability and spheroid formation. We incubated MSTO-211H-depleted cells and controls to increasing concentrations of abemaciclib (from 0.3 µM to 4.8 µM) and evaluated cell proliferation by MTS assay after 72-h treatment (**Figure 4B**). Abemaciclib did not significantly affect cell viability of RBL1/p107- and RBL2/p130-depleted MSTO-211H, as compared to control lines (MSTO-211H shRNA SCR), but reduced the viability of MSTO-211H with RB1/p105 depletion, suggesting that the other pocket proteins are able to mediate abemaciclib effects in absence of RB1/p105. We also tested whether pocket proteins can affect the capacity of DPM cells to form spheroids. However, as shown in **Figure 4C**, our results did not show any difference between controls or pocket protein-depleted MSTO-211H cells in their ability to form spheroids, with no detectable differences in dimension, shape, or number. In addition, the perimeter of necrotic and hypoxic cores had the same circumference (**Figure 4D**). Thus, our data suggest a functional overlap between RB proteins in this response (39).

### 3.5 Abemaciclib perturbs cell cycle progression and induces apoptosis in malignant pleural mesothelioma cell lines

**Figure 5:**
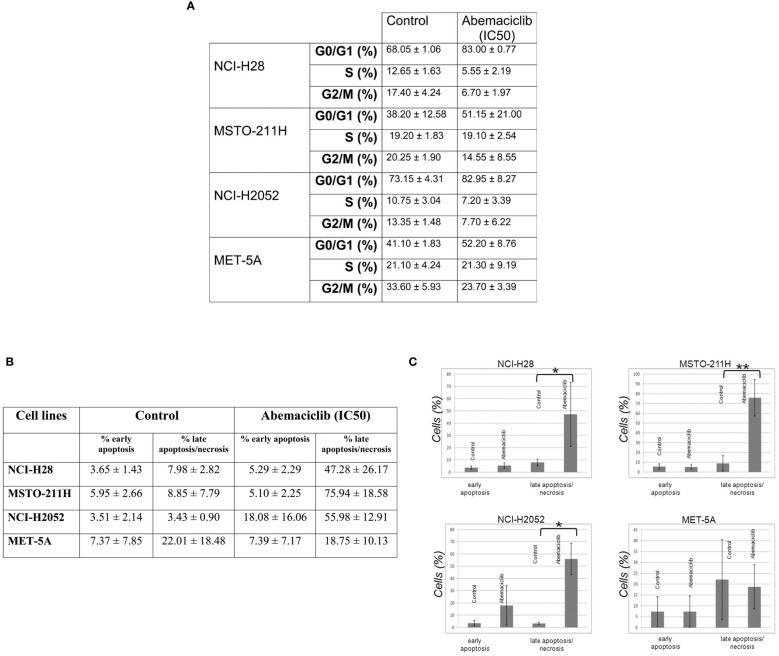
Abemaciclib effects on cell cycle progression and apoptosis induction in DPM cells. **(A)** The table reports the median ± SD of two independent FACS analyses of abemaciclib-treated DPM cells. The data obtained 48 h after treatment are shown; DMSO alone was added to untreated control cells. **(B)** FACS analysis was performed to investigate apoptosis using cell staining for Annexin V-FITC and propidium iodide (PI) in NCI-H28, MSTO-211H, NCI-H2052, and MET-5A cells 72 h with IC50 values. **(C)** Histograms show means with standard deviations of at least two independent experiments. Statistically significant differences were evaluated by one-way repeated-measures ANOVA with Tukey’s post-test and indicated as follows: *p < 0.05; **p < 0.01. DPM, diffuse pleural mesothelioma; DMSO, dimethyl sulfoxide; FACS, fluorescence-activated cell sorting.

We assessed the effects of abemaciclib treatment on DPM cell cycle progression by evaluating cellular DNA content by fluorescence-activated cell sorting (FACS) analysis at 48 h. We analyzed the cell cycle profile of NCI-H28, MSTO-211H, NCI-H2052, and MET-5A cell lines treated at their IC50 values, and we observed an accumulation of cells in the G0/G1 phase. Our results are consistent with a cell cycle arrest associated with abemaciclib-dependent inhibition of RB family members’ phosphorylation. In particular, our data showed an increase of approximately 15% in the G0/G1 phase in NCI-H28 and MSTO-211H and slightly less in NCI-H2052 cells (**Figure 5A**). We also observed a reduction of the G2/M phase in all three cell lines (NCI-H28, MSTO-211H, and NCI-H2052) and a decrease in the S phase for NCI-H28 and NCI-H2052. MET-5A cell cycle profile showed an increase in the G0/G1 phase, although this event did not significantly affect cell viability.

To assess whether abemaciclib induces cell death and better clarify which pathway of cell death is affected, we performed cytofluorimetric Annexin V assays after 48-h treatment with abemaciclib (IC50). Our data showed that DPM cells presented with positive Annexin V staining upon treatment. The percentages of early (only Annexin V-positive cells) and late apoptosis (both Annexin V- and PI-positive cells) demonstrated that abemaciclib induced apoptosis in all DPM cell lines tested without affecting control MET-5 A cells (**Figures 5B, C**).

### 3.6 Abemaciclib synergizes with cisplatin and AKTi VIII in suppressing DPM cell viability

**Figure 6:**
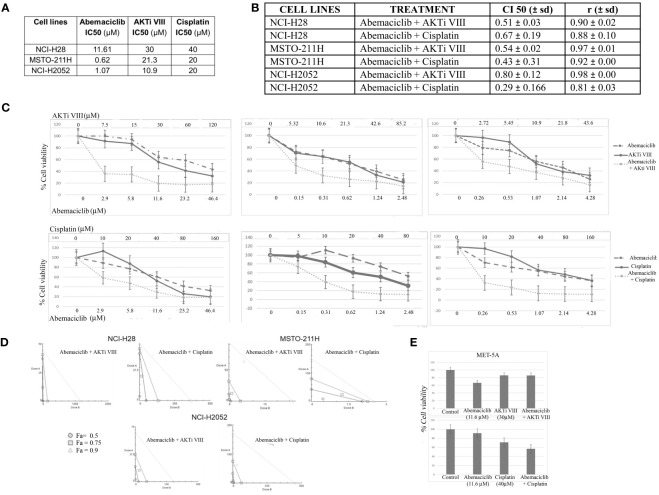
Synergistic effects of abemaciclib/AKTi VIII and abemaciclib/cisplatin combination on DPM cell lines. **(A)** Abemaciclib, AKTi VIII, and cisplatin IC50 values on DPM cell lines. These values were calculated from cell viability data obtained by MTS after 72 h of treatment with three compounds. **(B)** Table reporting the means ± standard deviations of combination index (CI) and r values of abemaciclib–AKTi VIII and abemaciclib–cisplatin combination at 50% of cell killing (CI50) following 72 h of treatment, calculated using the CalcuSyn software for each of two independent experiments. **(C)** Dose–response curves for abemaciclib alone, AKTi VIII, cisplatin alone, and combinations in NCI-H28, MSTO-211H, and NCI-H2052 cell lines at 72 h after treatment. Results represent the means of two independent experiments in triplicate and are expressed as percentages of cell viability over control cells treated with DMSO alone. **(D)** Isobologram analysis to assess synergism between abemaciclib–AKTi VIII and abemaciclib–cisplatin. Isobolograms are derived from the mean values of the dose–response experiments reported in panel C, through the CompuSyn software at fixed effect levels [fraction affected (Fa)] of 50%, 75%, and 90%. The points below the lines indicate synergism. **(E)** Histogram showing that 72 h of treatment with abemaciclib–AKTi VIII and abemaciclib–cisplatin at indicated combination doses had no toxic effect on immortalized normal mesothelial cells (MET-5A), as determined by MTS assay. Results are reported as means of two independent experiments and expressed as percentages of cell viability compared to control cells treated with DMSO alone. DPM, diffuse pleural mesothelioma; DMSO, dimethyl sulfoxide.

Next, we examined possible synergistic effects of abemaciclib in combination with either cisplatin, which is the first-line treatment against DPM, or a commercial AKT inhibitor, AKTi VIII, which we have shown to induce apoptosis in DPM cell lines (33). We treated five DPM cell lines for 72 h with the two agents both alone and in combination at five different concentrations in a constant ratio. The agents were added in twofold serial dilutions of both IC50 values (as determined in our previously published data) (33) In particular, based on IC50 values previously determined at 72 h of cisplatin and AKTi VIII (33, 36) (**Figure 6A**), we treated the DPM cell lines for 72 h with the three drugs, both alone (abemaciclib, cisplatin, and AKTi VII) and in combination at various concentrations in a constant ratio. The specific doses used and the percentages of cell viability are shown in **Figure 6C** and **Supplementary Figure 1B**. In addition, we evaluated the synergism by isobologram analysis, as shown in **Figure 6D**. Indeed, the Chou–Talalay method analysis revealed CI values <1 for all DPM cell lines tested (**Figure 6B**). To rule out possible cytotoxic effects of this drug combination on non-neoplastic cells, we treated the MET-5A with abemaciclib–AKTi and abemaciclib–cisplatin combination doses, which corresponded to the two-drug concentrations leading to a ~50% reduction in the viability of the NCI-H28 cells that are the two higher doses combined, as shown in the graphs (**Figure 6E**). Additionally, we observed no toxic effect of these drug combinations on MET-5A at 72 h following treatment as assessed by MTS assays (**Figure 6E**).

### 3.7 Abemaciclib alters cell viability of cisplatinresistant malignant pleural mesothelioma cells

**Figure 7:**
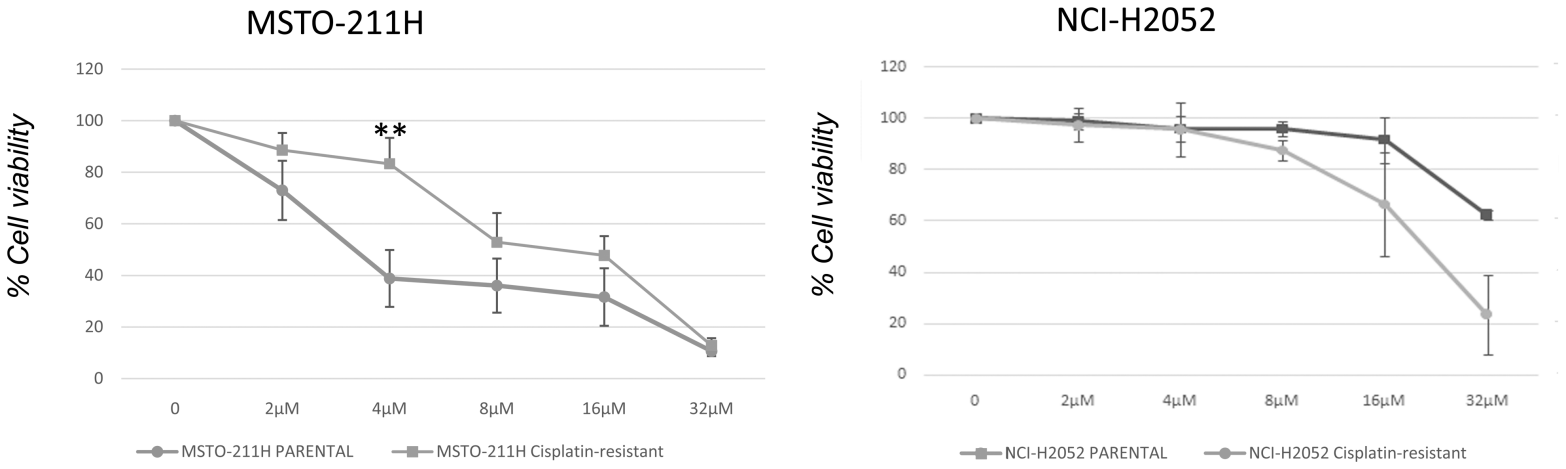
Effect of abemaciclib on NCI-H2052 and MSTO-211H cisplatin-resistant cell viability. Dose–response growth curves with different doses of abemaciclib determined by MTS assay after 72 h of treatment of MSTO-211H and NCI-H2052 cisplatin-resistant cell. Statistical analysis was carried out by unpaired Student’s t-test. Statistical significance was set at **p < 0.005 vs. control. Data are presented as mean ± standard deviation expressed as percentages of cell viability (calculated with respect to control cells treated with DMSO alone) of three independent experiments (n = 3). DMSO, dimethyl sulfoxide.

We next examined whether DPM cell lines resistant to cisplatin were sensitive to abemaciclib. We first generated a model of acquired resistance to cisplatin by repeating five cycles of 72-h treatments with increasing cisplatin doses. Cell resistance to cisplatin was evaluated by MTS at the end of treatment (data not shown). Then, cisplatin-resistant MSTO-211H and NCI-H2052 cells were challenged with different doses of abemaciclib, and interestingly, both DPM cell lines were sensitive to abemaciclib, suggesting that the new CDKi could be considered as a promising antitumoral drug for patients bearing cisplatin-resistant DPM (**Figure 7**).

## 4 Discussion

DPM is a very aggressive tumor, is poorly responsive to current therapy, and has a median survival of 9–17 months from diagnosis (40, 41). In addition to asbestos exposure, DPM development depends mostly on the inactivation of tumor suppressors such as *NF2*, *BAP1*, and *CDKN2A*, the loss of which recapitulates DPM features also in mice (14, 42, 43). Unfortunately, tumor suppressors are not good targets compared to oncogenic drivers. However, the recent discovery and successful clinical application of selective CDK4/6i able to restore RB canonical cell cycle restraining functions has provided another tool for promising targeted therapy approaches (20).

Here, we first aimed to assess the efficacy of first-generation CDK4/6i, ribociclib, palbociclib, and abemaciclib, on our panel of DPM cell lines characterized by the loss of *CDKN2A*. As expected, all CDK4/6i showed efficacy in reducing DPM cell viability, whereas normal immortalized MET-5A mesothelial cells were not affected by palbociclib at the range of concentrations tested or affected by abemaciclib and ribociclib at higher doses compared to those inhibiting cancer cells. The values of palbociclib affecting DPM cell lines had more variation between studies as already observed by Kratzke (29) in comparison with the studies of Bonelli (26), and Aliagas and Paternot as well (27, 28). Abemaciclib was the most effective CDKi on our panel of DPM cells, in line with data from Paternot (28), who analyzed by MTT the same drugs at a lower dose range on a similar cell panel (excluding ISTMES-2 and DPMB); Terenziani, who treated MSTO-211H and NCI-H28 with 0.5 µM abemaciclib; or Aliagas, who used a nanomolar drug range (27). Consistent with our results, MSTO-211H was the more sensitive cell line in all three independent analyses.

Based on initial observation, we then focused on abemaciclib and showed that treatment of NCI-H28, MSTO-211H, NCI-H2452, and NCI-H2052 at the IC50 prevented colony formation, indicating prolonged inhibition of DPM cell proliferation, thereby suggesting a permanent cell cycle withdrawal. These results recapitulate the data obtained by Aliagas et al., who also observed remarkable colony formation inhibition in the same cell line panel upon treatment with fixed doses (not the IC50) of abemaciclib and palbociclib. Similarly, Terenziani et al. (30) showed that prolonged abemaciclib treatment was sufficient to inhibit permanently NCI-H28 proliferation, although MSTO-211H resumed colony formation upon drug removal (30). This is different from our study in which both cell lines showed irreversible growth inhibition following drug treatment.

In DPM and particularly in pleural DPM, 20% of histological preparations show tumor growth into the lung parenchyma from the periphery in a complex 3D pattern (44, 45). These 3D cultures show differences in gene expression and response to therapy when compared to 2D models (44, 45). Therefore, to assess whether abemaciclib affected the growth of DPM cells grown in 3D cultures, we tested DPM cell lines for their ability to grow as both single and multiple spheroids. The formation of single spheroids was inhibited when abemaciclib was added at the time of plating (time 0) or 2 days after spheroid formation. These data suggest that abemaciclib can reach target cells deeply inside the 3D models. Consistently, when abemaciclib was administered to fully formed multiple spheroids, all DPM cells were not able to form second-generation 3D structures upon spheroid disaggregation and replating. Again, these data support an irreversible cell cycle exit of DPM cells upon treatment with this CDK4/6i in a model that better recapitulates *in vivo* tumor growth.

CDK4 and CDK6 are key factors for initiating cell proliferation by inactivating RB proteins by phosphorylation. Briefly, during quiescence, non-phosphorylated RB1/p105 directly binds and inhibits E2F transcription factors, which are crucial regulators of cell cycle genes. Upon mitogenic signaling, cyclin D increases and activates CDK4/6, which in turn phosphorylates and inactivates RB1/p105, resulting in E2F activation and cell cycle entry (46, 47).

Thus, to test abemaciclib action in modulating RB family members, we analyzed CDK4/6-mediated phosphorylation of RB family members, which have both distinct and redundant functions (48). As expected, CDK4/6-mediated phosphorylation at specific residues, such as S780 of RB1/p105, S780 of RBL1/p107, or S952 of RBL2/p130, markedly decreased in all DPM cell lines. Interestingly, the total levels of RB1/p105 were also affected in all cell lines, consistent with data from Terenziani (30). The total levels of RBL1/p107 decreased particularly in NCI-H28 and NCI-H2052, a mechanism possibly dependent on its direct transcriptional repression by the reactivated function of RBL2/p130. On the contrary, RBL2/p130 levels were only minimally affected by abemaciclib treatment, indicating interesting differences between RB family members. Notably, abemaciclib inactivates AKT kinase in lung cancer (38), and Terenziani et al. (30) observed that abemaciclib, alone or in combination with chemotherapy, reduced AKT activation in DPM cells. Accordingly, we observed a remarkable reduction in AKT S473 phosphorylation in DPM cells, suggesting that abemaciclib counteracts the pro-survival and anti-apoptotic oncogenic functions of AKT, which is highly activated in DPM (33). Consistent with abemaciclib-dependent AKT inactivation, we observed a decrease in phosphorylation of S941 on RBL2, which we previously demonstrated is indeed an AKT substrate. The concomitant reduction of RBL2/p130 phosphorylation on S952 and S941 likely contributes to restoring the nuclear tumor suppressor function of RBL2/p130, which acts as a member of the DREAM transcriptional complex coordinating cell fate decisions (49).

To assess the impact of the different RB proteins on DPM cell response to abemaciclib, we depleted by shRNA approaches each family member in MSTO-211H cells, which were then treated with a range of drug doses. Single silencing of each RB family member showed cell viability similar to untreated control cells, suggesting compensatory roles of other family members in DPM cell response to abemaciclib. Notably, depletion of any RB family member had no effect on DPM cell ability to form spheroids, indicating that either RB proteins do not play an important role in the 3D growth of DPM cells, or they are able to complement each other’s role (50). It would be interesting to simultaneously target different pocket proteins in DPM cells, which would allow us to dissect any specific contribution of RB family members in 2D and 3D cultures.

When we looked at the cell cycle distribution of DPM cells treated with abemaciclib at their respective IC50 values, we observed a remarkable increase in the G0/G1 phase and a reduction of the G2/M phase in all the three cell lines (NCI-H28, MSTO-211H, and NCI-H2052). Notably, NCI-H28 and NCI-H2052 also showed a decrease in the S phase. A significant growth arrest in G0/G1 was also observed by Terenziani in NCI-H28 and MSTO-211H upon treatment with 0.5 µM abemaciclib (30), confirming the effect of abemaciclib in modulating cell cycle distribution of DPM cells.

We evaluated the ability of abemaciclib to induce DPM cell death and showed that abemaciclib at IC50 value significantly increased the late apoptosis/necrosis fraction in DPM cell lines but not in normal mesothelial cells. The results are somewhat different from those reported by Terenziani et al. (30) and Aliagas and colleagues (27), who did not observe similar cell death of NCI-H28 and MSTO-211H treated with abemaciclib as a single agent. However, these differences can be easily attributed to different experimental approaches.

However, we can reasonably speculate the role of abemaciclib in inducing cell death pathways, considering the strong effect of abemaciclib in inhibiting AKT activation, which is pivotal for protecting cancer cells from apoptosis (33). Notably, the effect of abemaciclib on AKT activation is not observed after palbociclib treatment, which instead increases AKT S473 phosphorylation in DPM cells (26). This difference between the effects of abemaciclib and palbociclib on AKT activation has substantial importance for therapy, considering that DPM cells are highly dependent on active AKT pathways (33). In addition, our data showed that abemaciclib treatment by inhibiting pAKT affects also the mTOR signaling cascade, which is implicated in the abnormal growth and survival of cancer cells (51). Thus, abemaciclib alters the mTOR kinase pathway by downregulating p70 S6 kinase phosphorylation, which likely contributes to its significant anticancer activity in DPM.

AKT inhibition upon abemaciclib treatment could be also, at least in part, responsible for the strong inhibition of DPM cell growth observed in 3D (52, 53).

In this study, we also showed that abemaciclib synergized with cisplatin, consistent with the work of Terenziani et al. (30), who showed that combined treatment with abemaciclib enhanced the efficacy of cisplatin or pemetrexed and triggered stronger inhibition of cell proliferation while reducing AKT phosphorylation (30). Additionally, we showed that abemaciclib synergized with the selective AKT inhibitor AKTi VIII, thereby further supporting the rationale of this drug combination.

Previously published work (26–28) demonstrated that palbociclib induced G0/G1 arrest and senescence (54), consistent with other data supporting the action of CDK4/6i in inducing senescent-like phenotypes (54, 55). In addition to senescence, palbociclib induced autophagy, but these differences were cell context-dependent (30). As RB proteins are key regulators of senescence (56), it will be important to dissect in the future their specific role upon CDK4/6i treatment as well the effect of induced senescence-associated secretory phenotype on DPM micro/macroenvironment.

Because DPM often shows resistance to currently approved chemotherapy regimens (57), we developed a novel model of acquired resistance to cisplatin in MSTO-211H and NCI-H2052 DPM cells and demonstrated that abemaciclib effectively reduced cell viability of cisplatin-resistant DPM cells, suggesting that this CDK4/6i could be effective in the context of chemotherapy resistance.

Overall, our data identify abemaciclib as a very effective drug in inhibiting DPM growth in 3D and demonstrated its effect in combination with cisplatin and AKT inhibition in overcoming cisplatin resistance. We also show that RB family members can likely play a redundant role in modulating response to abemaciclib. Consistent with the recent identification of additional targets of abemaciclib (54), we demonstrated that abemaciclib induces a significant downregulation of the active AKT form, which likely impacts mesosphere formation, thereby suggesting that this CDK4/6i can synergize with AKT inhibitors to improve DPM therapy.

## Data availability statement

The datasets presented in this study can be found in online repositories. The names of the repository/repositories and accession number(s) can be found below: https://zenodo.org/uploads/11611146.

## Ethics statement

Ethical approval was not required for the studies on humans in accordance with the local legislation and institutional requirements because only commercially available established cell lines were used.

## Author contributions

AuC: Investigation, Methodology, Writing – review & editing. IF: Conceptualization, Formal analysis, Investigation, Methodology, Writing – original draft. FP: Conceptualization, Funding acquisition, Writing – review & editing. CI: Methodology, Writing – review & editing. LA: Methodology, Writing – review & editing. FC: Methodology, Writing – review & editing. RC: Methodology, Writing – review & editing. AIC: Methodology, Writing – review & editing. CV: Methodology, Writing – review & editing. RD: Methodology, Writing – review & editing. MQ: Methodology, Writing – review & editing. MD: Funding acquisition, Writing – review & editing. AM: Conceptualization, Writing – review & editing. AG: Conceptualization, Funding acquisition, Supervision, Writing – review & editing.

## Supplementary material

Supplementary Figure 1 Synergistic effects of abemaciclib/AKTi VIII and abemaciclib/cisplatin combination on DPM epithelioid cell lines. **(A)** Analysis of phosphorylated levels of pRB1/p105 (Ser 780) in NCH-H2452 and IST-MES 2 cell lines upon abemaciclib treatment. GAPDH was used as loading control. **(B)** Dose-response curves for abemaciclib alone, AKTi VIII, cisplatin alone and combinations in NCI-H2452 and IST-MES 2 cell lines at 72 h after treatment. Results represent the means of two independent experiments in triplicate, and are expressed as percentages of cell viability over control cells treated with DMSO alone.
