# Supplementary figures and images for: Pharmacological inhibition of CDK4/6 impairs diffuse pleural mesothelioma 3D spheroid growth and reduces viability of cisplatin-resistant cells
Source: Front Oncol. 2024 Jul 1;14:1418951. doi: 10.3389/fonc.2024.1418951 (PMC11246887; doi:10.3389/fonc.2024.1418951)

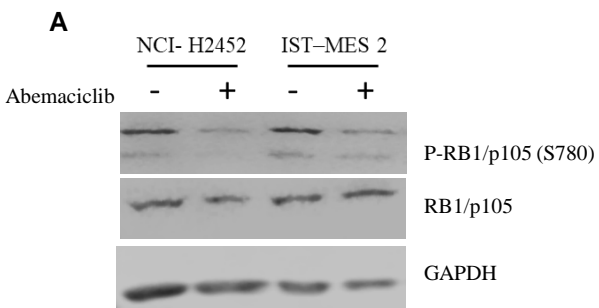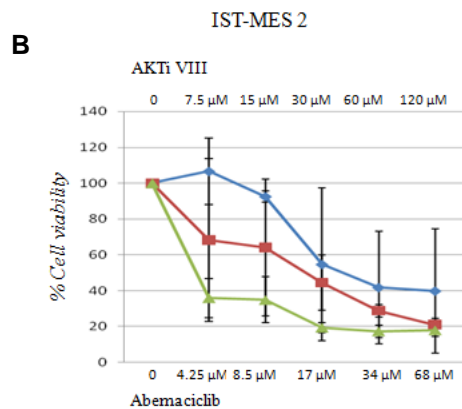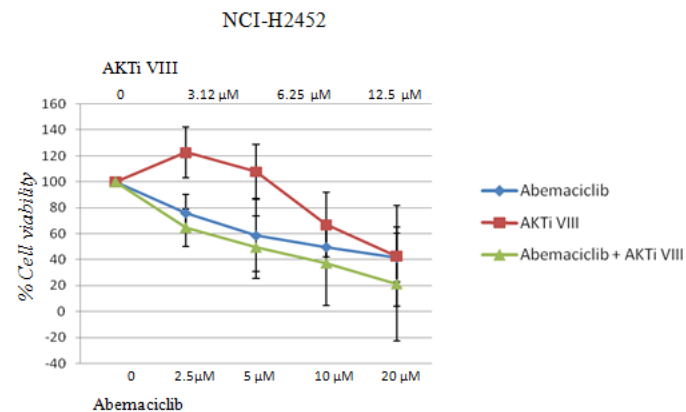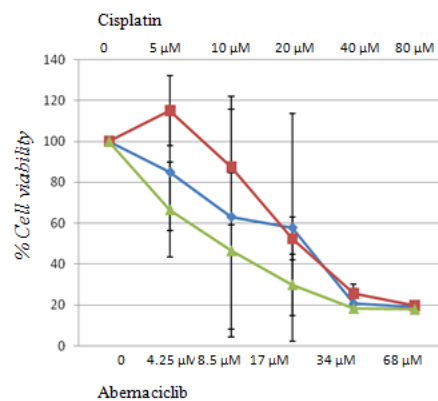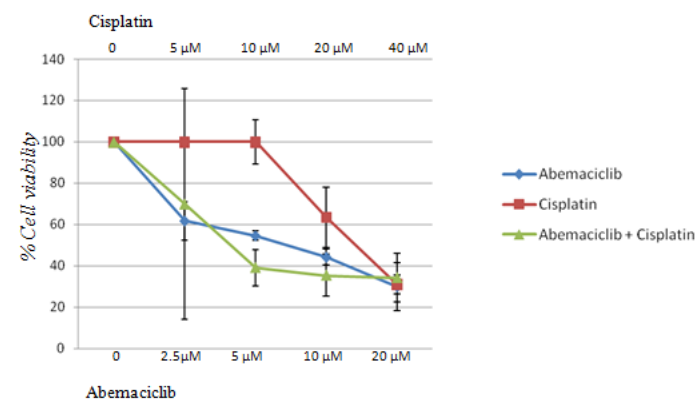

Supplement: Supplementary Figure 1 — Synergistic effects of abemaciclib/AKTi VIII and abemaciclib/cisplatin combination on DPM epithelioid cell lines. (A) Analysis of phosphorylated levels of pRB1/p105 (Ser 780) in NCH-H2452 and IST-MES 2 cell lines upon abemaciclib treatment. GAPDH was used as loading control. (B) Dose-response curves for abemaciclib alone, AKTi VIII, cisplatin alone and combinations in NCI-H2452 and IST-MES 2 cell lines at 72 h after treatment. Results represent the means of two independent experiments in triplicate, and are expressed as percentages of cell viability over control cells treated with DMSO alone. [file DataSheet_1.pdf]
